# Development of a Computerized Adaptive Test for Schizotypy Assessment

**DOI:** 10.1371/journal.pone.0073201

**Published:** 2013-09-03

**Authors:** Eduardo Fonseca-Pedrero, Luis Fernando Menéndez, Mercedes Paino, Serafín Lemos-Giráldez, José Muñiz

**Affiliations:** 1 Department of Educational Sciences, University of La Rioja, La Rioja, Spain; 2 Department of Psychology, University of Oviedo, Oviedo, Spain; 3 Center for Biomedical Research in the Mental Health Network (CIBERSAM), Madrid, Spain; Population Health and Preventive Medicine, Malaysia

## Abstract

**Background:**

Schizotypal traits in adolescents from the general population represent the behavioral expression of liability for psychotic disorders. Schizotypy assessment in this sector of population has advanced considerably in the last few years; however, it is necessary to incorporate recent advances in psychological and educational measurement.

**Objective:**

The main goal of this study was to develop a Computerized Adaptive Test (CAT) to evaluate schizotypy through “The Oviedo Questionnaire for Schizotypy Assessment” (ESQUIZO-Q), in non-clinical adolescents.

**Methods:**

The final sample consisted of 3,056 participants, 1,469 males, with a mean age of 15.9 years (*SD* = 1.2).

**Results:**

The results indicated that the ESQUIZO-Q scores presented adequate psychometric properties under both Classical Test Theory and Item Response Theory. The Information Function estimated using the Gradual Response Model indicated that the item pool effectively assesses schizotypy at the high end of the latent trait. The correlation between the CAT total scores and the paper-and-pencil test was 0.92. The mean number of presented items in the CAT with the standard error fixed at ≤0.30 was of 34 items.

**Conclusion:**

The CAT showed adequate psychometric properties for schizotypy assessment in the general adolescent population. The ESQUIZO-Q adaptive version could be used as a screening method for the detection of adolescents at risk for psychosis in both educational and mental health settings.

## Introduction

Schizotypy is a complex construct which is intimately related at the historical, genetic, neurodevelopmental, neurocognitive, social, emotional and psychophysiological levels to psychotic disorders [1]–[3]. Independent follow-up studies indicate that adolescents from the general population who report schizotypal experiences such as magical thinking, hallucinatory experiences, delusional ideation and/or anhedonia have a greater risk of transiting toward schizophrenia-spectrum disorders [4]–[9]. Also, the subclinical expression of the psychosis phenotype has been associated with the same risk factors related to schizophrenia (e.g., cannabis use, urbanicity, trauma, etc.) conferring aetiological validity on this construct and suggesting a possible continuity between the clinical and the subclinical psychosis phenotypes [10]. In this sense, schizotypal traits in adolescence might represent the behavioral expression of vulnerability for psychotic disorders in the general population [11], [12].

A current line of research in the psychosis field is based on the idea that early detection, prevention and intervention in young people at risk for psychosis could mitigate or reduce the impact the disorder can cause on personal, familial, work and social spheres [13], [14]. Among other aspects, this fact has driven the construction and validation of measurement instruments for the assessment of schizotypy at early ages [15]. The aim of the psychometric high-risk paradigm is the detection, by means of self-reports and based on their score profiles, of those participants at risk for psychosis in the future [16], [17]. This paradigm is considered a reliable, valid and useful method for the psychometric detection of individuals at risk for psychosis. In comparison to other techniques, the use of these tools constitutes a rapid, efficient and noninvasive method of assessment. Moreover, it allows the study of symptoms that are similar to those found in patients with schizophrenia while avoiding the confounding effects frequently found in these individuals (e.g., medication or stigmatization) [6], [17], [18].

The detection of these types of individuals at risk for psychosis, whether in clinical or educational settings, requires having adequate measurement instruments that allow us to make solid and well-founded decisions based on the data. The Wisconsin Schizotypy Scales [19] and the Schizotypal Personality Questionnaire [20] are among the most utilized tools in the literature for the assessment of this construct in adult populations. Likewise, and given that adolescence is a developmental period of special risk for psychosis and related disorders [21], [22], efforts have also been directed at the assessment of schizotypal experiences and traits in this age group. Good examples of these self-reports are the Junior Schizotypy Scales [23], or The Oviedo Schizotypy Assessment Questionnaire (ESQUIZO-Q) [24]. The cited self-reports have presented adequate psychometric properties under Classic Test Theory (CTT); however, it is necessary to rigurously advance in the assessment of this construct with the incorporation of psychometric advances such as differential item functioning, measurement invariance across age or cultures [25], [26] or Item Response Theory (IRT) [27].

IRT originates as a complementary psychometric model and offers many advantages over CTT [28], [29]. The IRT approach along with computer implementation has changed the psychological assessment panorama with new forms of measurement emerging, such as Computerized Adaptive Testing (CAT) [30]–[32]. In this regard, an interesting expansion has taken place as shown by the CATs constructed in diverse areas as education (e.g., English) [33], health (e.g., quality of life) [34], [35] or psychopathology (e.g., depression, personality) [36], [37]. CAT involves the computerized administration of a test in which each item is dynamically selected from a pool of items until a pre-specified measurement precision is reached. CAT successively selects items in order to maximize the precision of the measurement instrument based on what is known about the person from previous items. The essential idea is that when adjusting the items to the competency (or latent trait) of the test taker, once these are calibrated according to an IRT model far fewer items are needed to assess individuals with precision in comparison to paper-and-pencil tests. Thus, items and time are saved through the use of precision and efficiency. In addition, this way of proceeding further motivates the assessed subjects as the items they must respond to are neither too easy nor too difficult, but rather are adjusted to their latent-construct level [38]. Moreover, through IRT, an Item Characteristic Curve (ICC) is constructed for each item. This curve, or trace line, reflects the probability of the person’s response to each item and his/her level on the latent construct (e.g., schizotypy) measured by the scale. Furthermore, IRT allows us to estimate the contribution each item makes to the assessment for each level of the latent construct: the Information Function. Standard error is inversely linked to the information distribution used, and hence, the estimated standard error is available for each assessment.

To date, a CAT for schizotypy assessment in the general adolescent population has not been developed. Advances in the measurement field, along with IRT, improve the precision in the evaluation of this construct as well as its comprehension. Likewise, the incorporation of more robust statistical models may allow improvement in the detection procedures for this group of participants at psychometric risk for psychosis. Within this research context, the main goal of this study was to analyze the psychometric properties and efficiency of a hypothetical CAT to assess schizotypy, via ESQUIZO-Q, in a representative sample of adolescents from the general population. Its psychometric quality and efficiency was examined in its two modalities, both for a polytomous response format as well as a dichotomous response format, comparing the estimates obtained with the classic paper-and-pencil test to those obtained with the CAT.

## Methods

### Participants

Two stratified random cluster samplings were carried out at the classroom level in a population of approximately 37,000 students selected from the Principality of Asturias (a region in northern Spain) during two academic years (2008–2009 and 2009–2010). Other data from this research has been published elsewhere [24], [39]. The students were from various public and state-subsidized secondary schools and vocational training centres, as well as from a wide range of socio-economic levels. The strata were created on the basis of geographical zone (East, West, Centre and Mining area) and educational stage (compulsory – to age 16– and post-compulsory). The likelihood of a school being included was directly proportional to the number of students in it. The final sample was made up of N = 3,056 participants, 1,496 boys (48.1%). The mean age was 15.9 years (*SD* = 1.2), with an age range of 14 to 18 years. The sample distribution according to age was the following: 14 year olds (N = 400; 13.1%), 15 year olds (N = 780; 25.5%), 16 year olds (N = 885; 29%), 17 year olds (N = 703; 23%) and 18 year olds (N = 288; 9.4%).

### Instruments

*The Oviedo Schizotypy Assessment Questionnaire* (ESQUIZO-Q) [24] is a self-report composed of 51 items in a 5-point Likert-type response format (1 = “*completely disagree*”; 5 = “*completely agree*”) designed to assess schizotypal traits and experiences in adolescents. The ESQUIZO-Q is based on the diagnostic criteria proposed in the DSM-IV-TR [40] and on Meehl’s schizotaxia model [41] regarding genetic predisposition to schizophrenia. The ESQUIZO-Q comprises a total of 10 subscales: Ideas of Reference, Magical Thinking, Unusual Perceptual Experiences, Odd Thinking and Language, Paranoid Ideation, Physical Anhedonia, Social Anhedonia, Odd Behavior, Lack of Close Friends and Excessive Social Anxiety. These subscales are grouped into three general dimensions: Reality Distortion, Anhedonia, and Interpersonal Disorganization. The psychometric properties of the ESQUIZO-Q have been extensively analyzed under CTT. The general schizotypy dimensions have been empirically derived through exploratory factor analyses and a rigorous process of item treatment according to international standards. No item in the ESQUIZO-Q showed differential functioning for gender. Internal consistency levels for the ESQUIZO-Q subscales ranged from 0.62 to 0.90 [39], [42]. Items of the ESQUIZO-Q are available as Supporting Information S1.

*The Oviedo Infrequency Scale* (INF-OV) [43] is a 12-item self-report with a 5-point Likert-type rating scale format (1 = “*completely disagree*”; 5 = “*completely agree*”). Its goal is to detect participants who respond randomly, pseudorandomly or dishonestly on self-reports (e.g., *“The distance between Madrid and Barcelona is greater than between Madrid and New York”*). This type of self-report is frequently used in studies on psychosis proneness in both clinical and non-clinical populations. Once the answers were dichotomized, students with more than 2 incorrect responses on this self-report were removed from the study. A total of 94 participants presented a score above two points on the INF-OV. No statistical differences were found between the group of participants who obtained more than two points on the INF-OV and the final sample.

### Ethic Statement

Written informed parental/guardian consent was obtained for all minors involved in the study. The study is part of a wider investigation on the detection and early intervention in psychological disorders in adolescence and was approved by the Research and Ethics Committees at the University of Oviedo and the Department of Education of the Principality of Asturias.

### Procedure

The administration of the questionnaires was conducted in a collective manner in groups of 10–35 students during the school schedule and in a room prepared for this purpose. The study was presented to participants as an investigation regarding diverse personality characteristics, assuring participants of the confidentiality of their answers as well as the voluntary nature of their participation. The completion of the questionnaires was conducted under the supervision of a researcher at all times.

### Data Analyses

First, the descriptive statistics for the ESQUIZO-Q subscales and second-order dimensions were calculated under CTT. Then, the calibration of the parameters of the 51 items in the ESQUIZO-Q under IRT was performed. For this purpose, Samejimás Graded Response Model (GRM) [44] and the two parameter logistic model (2PL) [45] were used. For the psychometric calibration of the ESQUIZO-Q items, several analyses were conducted: a) unidimensionality; b) local independence; c) monotonicity; d) model fit of GRM; and e) DIF by gender. In regard to the unidimensionality of the ESQUIZO-Q items, it can be assumed that the original 51-item pool is not unidimensional. This fact will negatively influence the psychometric functioning of the CAT, which is based on the unidimensionality assumption. In this sense, several analyses were performed using the item bank. First, 51 items were calibrated, and second, 9 items and then 2 items were removed successively and iteratively depending on the fit to the data, the standard error estimation and inspection of the ICC. From a psychometric viewpoint, it is interesting to analyze test scores under a dichotomous-type and Likert format as it can be observed that the number of response categories of items affects the metric properties of the measurement instruments [46], [47]. At the same time, in psychopathology measures, the response format that is more frequently used is the dichotomous type. The study of both types of formats allows us to make comparisons and observe levels of precision under CTT and IRT. In this study, the Likert format responses were dichotomized once the ESQUIZO-Q was administered (values 1–3 codified as “0” and values 4–5 as “1”). The value *θ* (theta) represents participantś scores on the latent construct (schizotypy), parameter *a* (or slope) is related to the items discriminative power and parameter *b* (or location) to item threshold or difficulty level. A steeper slope indicates a closer relationship to the construct and therefore a more discriminating item. The larger the location parameter, the more of the measured construct a respondent must have to endorse that item. In the GRM, the number of estimated *b* parameters is equal to the item response category number minus one. In an item with a Likert response format with five options, the number of estimated *b(k)* parameters would be four. Each *b(k)* parameter indicates the probability of stepping from a lower category to a higher category. The 2PL model is used to calibrate the items of a dichotomous nature (e.g., Yes/No). The GRM is a model of IRT for polytomous data, and is a generalization of the 2PL model. In the 2PL model, discrimination parameter *a* and difficulty parameter *b* can also be estimated. Given that it is utilized for dichotomous models, the estimated number of parameter *b* is one.

Finally, with respect to CATs, a simulation study was conducted. First, we used our own software with client-server technology for the implementation of two algorithms, one for polytomous data and another for dichotomous data [28]. The algorithm, which is common to both models, behaves in the following manner: 1) initially, we start from an estimated *b* parameter *(1)* which we assume as the population measure; 2) with the aim of estimating the following *θ* value, the item that provides the greatest information value for this *θ* is selected and is presented to the participant to be responded to; 3) once we obtain the response of the participant, it is used to obtain, through the Maximum Likelihood procedure [48], a new estimation of *θ* as well as the corresponding standard error estimation (*SE*); and 4) steps 2 and 3 are repeated until the *SE* value is below an established value, usually 0.30. The criteria *SE* = 0.30 corresponds to a reliability of *p* = 0.9 (*p* = 1-*SE* ^2^) [49].

Second, the study was performed using CAT in its two modalities, dichotomous and polytomous. Also, we used two stopping rule criteria (*SE* <0.30; *SE* <0.50) for illustrative purposes. The stopping rule used was the pre-specified level of measurement precision. The incorporation of the two stopping rule cut-offs allows us to examine the degradation effect in the functioning of the CAT. It is obvious that reliability decreases as the CAT uses a stopping rule with a higher standard error. The same items given by participants in the classic paper-and-pencil test were used as inputs. Two analyses were conducted: a) one of simulation calibrating the CAT items from the total sample of 3,056 cases to subsequently select a subsample of 500 random cases that served as inputs for the CATs; and b) an initial sample of 3,056 cases was subdivided into two random and independent subsamples, one of 500 cases that served as a sample for the administration of the CATs and another of 2,556 participants that was used to establish the estimated parameters of the CAT items. Extracting the 500 cases that were used for the administration of the CATs from the same sample used to calibrate the items (3,056 cases in our study) may lead to capitalization on chance providing a flattering outcome [36]. Although the initial sample we started with was sufficiently large to guarantee the representativeness of the item parameters of the CAT, we proceeded to conduct the study dividing the initial sample into two differentiated and totally independent parts. SPSS 15.0 [50], Parscale [51] and Multilog 7.0 [48] were used for statistical analyses.

## Results

### Descriptive Statistics

Table 1 shows the descriptive statistics for the subscales and general schizotypy dimensions of the ESQUIZO-Q in the total sample, under CTT. As can be observed, the levels of internal consistency of the subscales and second-order dimensions of the ESQUIZO-Q ranged from 0.61 to 0.87.

**Table 1:** Descriptive statistics for the subscales and the dimensions of the Oviedo Questionnaire for Schizotypy Assessment (ESQUIZO-Q).

Subscales	N° Items	*M*	*SD*	Asymmetry	Kurtosis	Range	AlphaCronbach
Ideas of Reference	4	6.16	2.61	1.42	2	4–20	0.70
Magical Thinking	5	7.69	3.04	1.36	1.88	5–25	0.67
Unusual Perceptual Experiences	7	10.38	4.36	1.84	4.06	7–35	0.79
Paranoid Ideation	5	8.10	3.31	1.3	1.77	5–25	0.74
Physical Anhedonia	4	7.82	2.64	0.7	0.61	4–20	0.60
Social Anhedonia	5	7.62	2.42	1.09	1.27	5–19	0.61
Odd Thinking and Language	6	13.96	4.71	0.39	−0.27	6–30	0.77
Odd behavior	4	6.93	2.88	1.27	1.7	4–20	0.68
Lack of Close Friends	4	9.57	3.71	0.39	−0.45	4–20	0.63
Excessive Social Anxiety	7	15.09	5.19	0.71	0.41	7–35	0.78
*Dimensions*							
Reality Distortion	21	32.34	10.39	1.39	2.76	21–97	0.84
Anhedonia	9	15.43	4.1	0.78	0.95	9–35	0.66
Interpersonal Disorganization	21	45.56	11.83	0.45	0.1	21–93	0.87

### Parameter Estimation Under IRT

Table 2 shows the 5 estimated parameters corresponding to GRM as well as the *λ* value of each item in this order. This value (λ = a/√(1+a^2^)) is interpreted as the degree of relationship that exists between *θ* (latent trait) and *S* (variable underlying the item). Likewise, the estimated values corresponding to the 2PL model can be observed. For example, within the 2PL model it can be seen that the item with *a* parameter was 50, whereas the item with the greatest *b* parameter was 41.

**Table 2:** Estimated parameters of the 51 items according to Samejima’s Graded Response Models (GRM) and the two-parameter logistic model (2PL).

	*GRM*					*2 PL*		
Items	*a*	*b*(1)	*b*(2)	*b*(3)	*b*(4)	λ	a*	b*
1	0.28	2.34	8.72	14.1	17.7	0.27	0.68	5.73
2	0.34	1.13	7.39	12	15.1	0.32	0.61	6.6
3	0.93	−0.74	0.65	1.94	3.26	0.68	1.24	1.28
4	0.68	−2	−0.1	1.6	4.01	0.56	1	0.9
5	1.04	0.74	2.24	3.13	4.21	0.72	1.35	2.28
6	1.06	0.83	2.11	3.11	4.12	0.73	1.25	2.44
7	1.49	0.92	1.64	2.04	2.82	0.83	1.72	1.54
8	075	1.12	2.39	4.14	5.85	0.6	1.07	2.78
9	0.7	−2.45	−0.4	1.56	3.67	0.57	1.19	0.75
10	0.71	0.24	1.77	3.26	5.17	0.58	2.03	2.03
11	1.18	0.41	1.53	2.96	3.9	0.76	1.43	2.25
12	1.45	0.26	1.06	1.94	2.86	0.82	1.78	1.39
13	0.42	2.34	7.14	10.4	12.1	0.39	0.81	5.25
14	1.07	0.56	1.7	2.69	3.64	0.73	1.65	1.69
15	0.75	−1.23	0	1.05	2.98	0.6	1.07	0.53
16	0.69	−2.5	−0.4	1.6	4.39	0.57	1.08	0.85
17	1.55	0.47	1.31	2.07	2.82	0.84	1.98	1.47
18	1.19	−0.91	0.25	1.39	2.64	0.77	1.94	0.75
19	0.71	−1.56	−0.1	0.91	2.55	0.58	0.91	0.91
20	0.75	−0.17	1	1.95	3.5	0.6	1.04	1.22
21	1.4	1.03	1.97	2.99	3.55	0.81	1.55	2.44
22	0.16	−3.44	3.44	14.1	18.2	0.16	0.22	10.4
23	1.16	−1.28	0	1.13	2.5	0.76	1.67	0.61
24	1.11	0	0.97	1.92	2.79	0.74	1.34	1.35
25	1.03	0.47	1.41	2.59	3.76	0.72	1.48	1.69
26	0.71	−1.4	−0.17	0.81	2.34	0.58	0.93	0.37
27	0.35	−0.1	3.99	7.91	10.43	0.33	0.55	4.85
28	2.14	1.29	1.91	2.39	2.76	0.91	2.23	1.97
29	0.95	0.05	1.26	2.29	3.69	0.69	0.21	1.62
30	198	1.24	1.85	2.33	2.87	0.89	2.69	1.66
31	0.86	1.4	0.19	1.84	3.66	0.65	1.37	1.02
32	2.32	1.2	1.96	2.45	2.87	0.92	2.94	1.84
33	1.07	−0.3	1.08	2.35	3.77	0.73	1.58	1.49
34	1.87	0.43	1.42	2.48	3.35	0.88	2.67	1.73
35	1.6	0.44	1.31	2.18	2.94	0.85	2.2	1.5
36	1.47	0.26	1.31	2.22	3.05	0.83	1.82	1.63
37	0.15	−7.7	0.58	10.63	15.09	0.15	0.16	9.87
38	1.74	0.51	1.47	2.26	2.93	0.87	2.11	1.69
39	1.65	0.35	1.19	1.8	2.56	0.86	1.8	1.38
40	0.34	1.26	7.3	10.54	12.72	0.32	0.47	7.43
41	0.18	−1.7	6.75	15.64	20.39	0.18	0.27	10.5
42	1.15	−0.4	1.04	2.28	3.64	0.75	1.61	1.51
43	0.27	3.84	10.81	15.47	17.47	0.26	0.41	10
44	1.27	0.82	1.71	2.54	3.4	0.79	1.41	2.02
45	1.62	0.6	1.33	1.89	2.73	0.85	1.76	1.47
46	1.34	0.57	1.76	3.23	4.25	0.8	1.72	2.39
47	1.91	0.92	1.86	2.68	3.21	0.89	2.03	2.23
48	1.28	0.58	1.66	2.51	3.57	0.79	1.67	1.78
49	1.7	0.57	1.16	1.62	2.35	0.86	1.83	1.22
50	1.93	1.1	1.91	2.63	3.29	0.89	3	1.77
51	1.54	1.53	2.4	3.3	3.88	0.84	2.25	2.26

**Note.** *a*: Item discriminative power according to the MRG; *b*(_k_): Item difficulty index; *λ*: (λ = a/√(1+a^2^)) degree of relationship between *θ* and the continuous variable underlying the item;

a*: item discrimination parameter;

b*: item location parameter.

### ESQUIZO-Q Computerized Adaptive Testing

**Figure 1:**
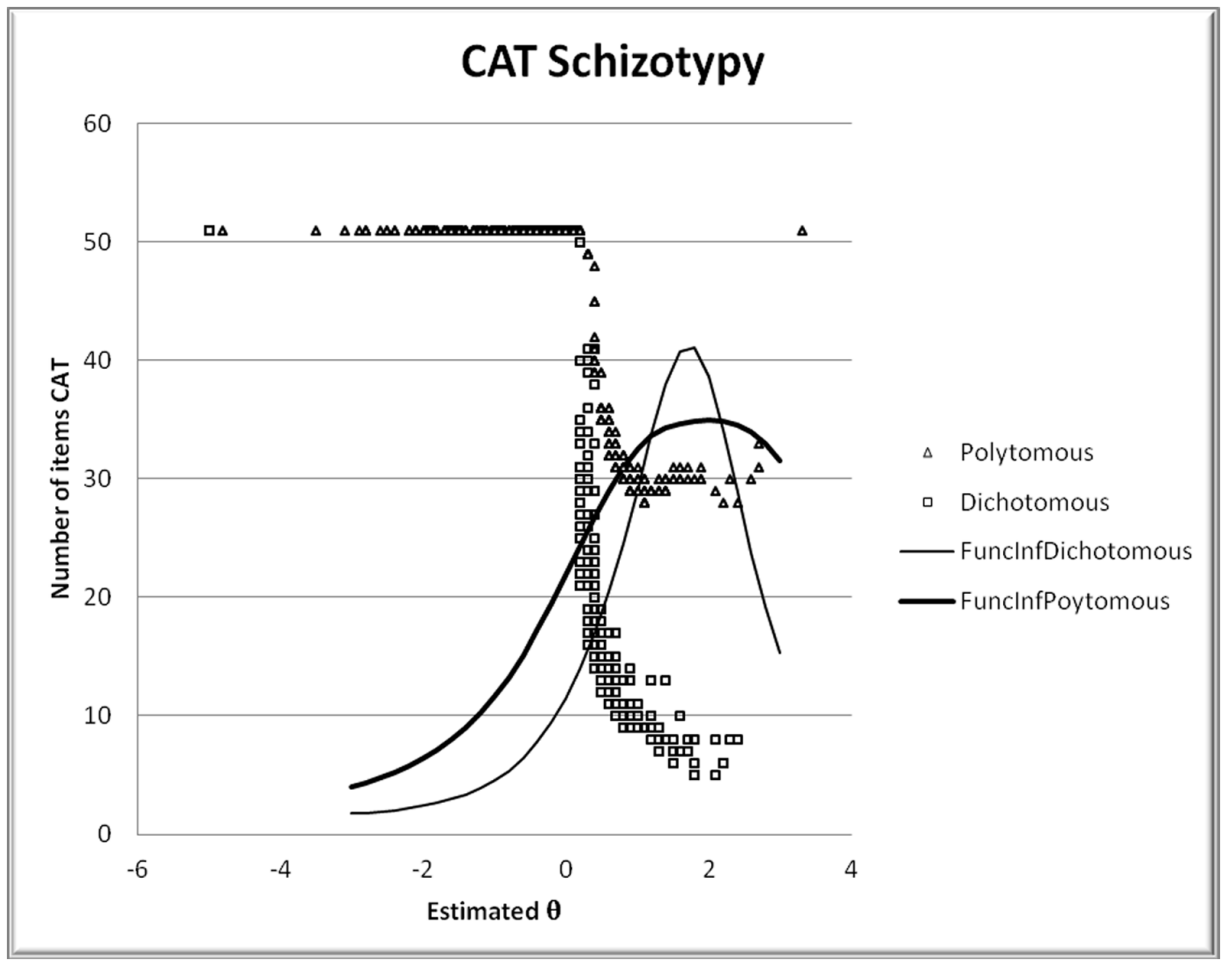
Information Functions of the item pool with polytomous and dichotomous data and number of items utilized in each CAT for the θ estimation.

The random subsample of 500 participants obtained from the initial sample of 3,056 participants was used to calibrate the 51 items of the CATs. The *θ* for each participant was estimated both for the polytomous and dichotomous CAT. In Figure 1, the Information Function that corresponds to both models used, as well as the estimated *θ* values in relation to the number of items presented to reach the cut-off criterion of *SE* <0.30 (stopping rule of the CAT) is shown. As can be seen in this Figure, the Information Function of the polytomous model provides more information for a greater value range than the dichotomous model. Both functions provide optimal estimations in those adolescents with high latent-trait values (schizotypy).

**Figure 2:**
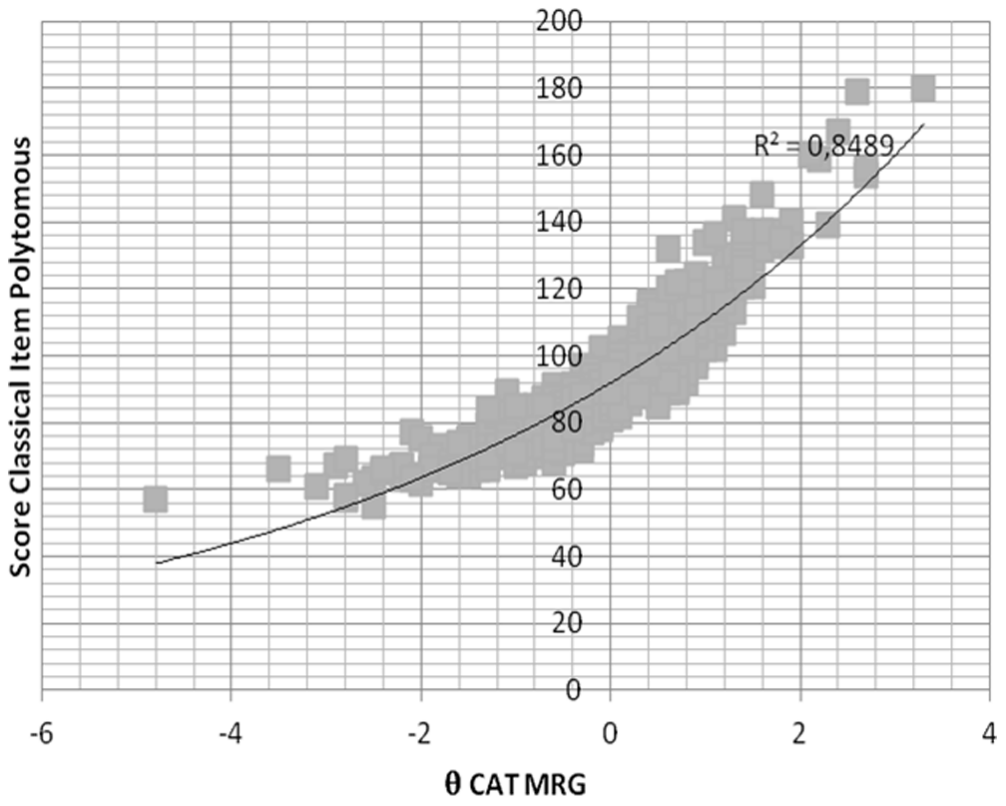
Correlation between the polytomous test total score and the score obtained with the polytomous CAT. Note: MRG: Graded Response Model.

**Figure 3:**
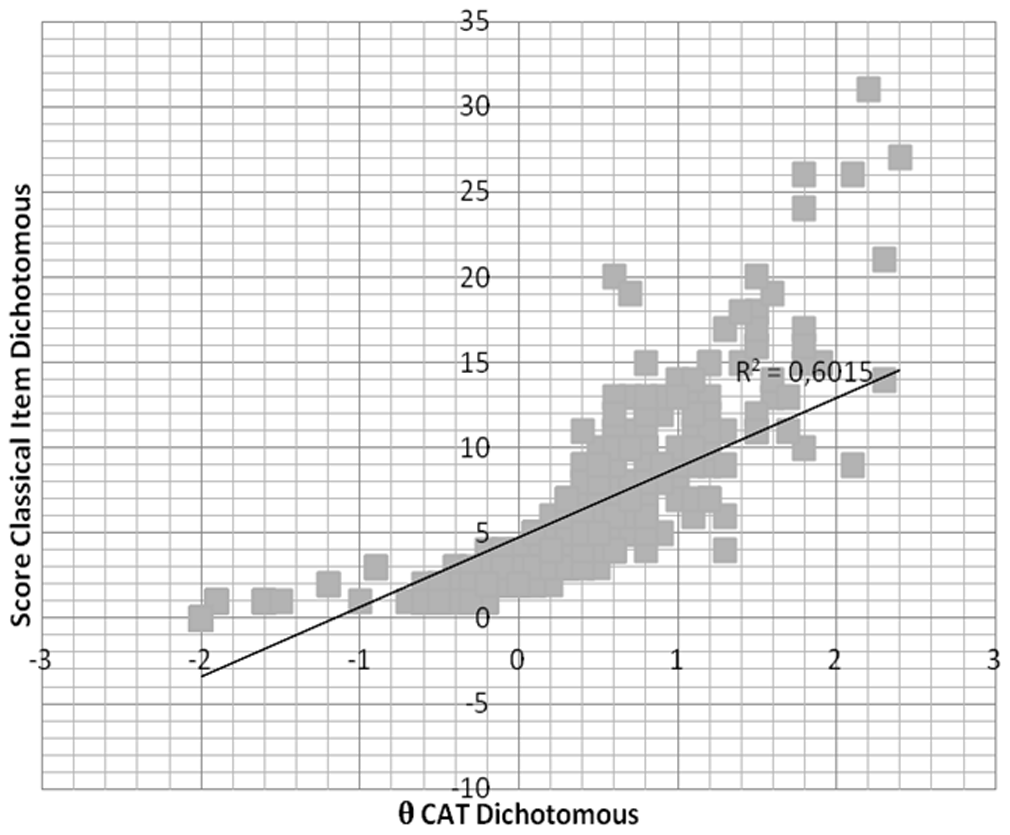
Correlation between the dichotomous total test score and the score obtained with the dichotomous CAT.

With the aim of checking the validity of the CATs, the total scores on the pencil-and-paper ESQUIZO-Q were correlated with the two modalities of the CAT. As can be observed in Figure 2, the correlations between the ESQUIZO-Q total score and the estimated *θ* for the polytomous CAT was 0.92. In the case of the dichotomous CAT (see Figure 3), the correlation between them was 0.88. It is worth mentioning that the value of the Pearson correlation between the ESQUIZO-Q polytomous and dichotomous total scores, according to CTT, was 0.82.

Table 3 shows the mean number of items used and the percentage of cases that exhaust the item pool of the CAT as a function of the stopping rule. In addition, the correlation between the CAT and the ESQUIZO-Q total score (pencil-and-paper version) is also presented in function of the response format (dichotomous and polytomous) and the two stopping rules (*SE* <0.30 vs. *SE* <0.50). The incorporation of the two stopping rule cut-offs allows us to examine the degradation effect in the functioning of the CAT. As can be observed, if the stopping rule adopted is *SE* <0.30, with the polytomous CAT, 199 cases (40%) of the 500 randomly selected obtain precise trait estimations with a number of items below 51. In the case of the dichotomous CAT, it rises to 303 cases of the 500 (60%). The mean number of necessary items to achieve a reliable estimation of the 199 cases with the polytomous CAT was 34±6 items, whereas the 304 cases of the dichotomous CAT require a mean number of 17±9 items. The correlation between the dichotomous and polytomous CAT was 0.60.

**Table 3:** Mean number of items used and percentage of items that exhaust the CAT item bank and correlation between the CAT and the complete test score in function of the type of CAT and stopping rule.

StoppingRule	CAT Items	Number of items used		% of CATs that exhaust the bank before reaching the stopping rule	Correlation CATand ESQUIZO-Q (pencil-and-paper) score
		*Mean* *	*SD* *		
*SE*(*θ*) <0.30	Polytomous	44 (34)	9 (6)	301 of 500 (60%)	0.92
	Dichotomous	30 (18)	19 (9)	197 of 500 (39%)	0.88
*SE*(*θ*) <0.50	Polytomous	18 (15)	12 (8)	42 of 500 (8.4%)	0.83
	Dichotomous	18 (15)	17 (14)	91 of 500 (18%)	0.58

**Note**. *SE* = Standard Error Estimation; *θ* = Latent trait.

* In parenthesis, the number of items used without taking into account those cases that exhaust the item pool before reaching the established cut-off criterion.

With the aim of optimizing the CAT, we proceeded to calibrate the item pool of the CAT disregarding 9 items that presented a poor fit to the data. These items were related to the Anhedonia dimension of the ESQUIZO-Q. The selection of these items was conducted attending, on the one hand, to the examination of the *SE* of the item parameters as well as to the visual inspection of the ICC. The correlation found between the polytomous 42-item CAT total score and its total score was 0.91. When two independent and random samples were utilized, one with 2,556 cases to calibrate the items, and the other with 500 cases to use as inputs in the CATs, and fixing the *SE* <0.30, the correlation between the ESQUIZO-Q and the 42-item polytomous CAT total scores decreased to 0.58. If we discard those cases in which the CAT exhausts the 51-item pool before reaching a reliable estimation (termination criterion), the correlation rises to 0.86. This disparity in the correlations with respect to the study, in which only one sample was used, may be attributed to the distortion of the item parameters as a consequence of the substantial reduction of 500 items from the initial sample.

With the objective of counteracting the effect that occurred previously due to the reduction of sample to calibrate the data, we proceeded to eliminate from the item pool all those items that, according to the visual inspection of their ICC and standard errors, presented a worse fit to the data. A total of 11 unfit items were eliminated and the item pool was once again calibrated, now with 40 items. The obtained results are similar to those obtained with the original 51 item pool and with a single sample. The correlation between the polytomous ESQUIZO-Q (paper-and-pencil-test) with the *θ* estimation with the polytomous CAT exhausting the item pool of 40 elements was 0.92, whereas that corresponding to the relationship conventional dichotomous test versus dichotomous CAT was 0.82. Likewise, when using the polytomous CAT, an average of 34±7 items were needed if the cases in which the 40 exhausted pool items are included, but only 27±5 of the 40, if we only consider those tests that achieve the established cut/off criterion.

## Discussion and Conclusions

The field of schizotypy assessment has considerably advanced in the last two decades. Also, there have also been numerous advances in the field of measurement as well as educational and psychological assessment [52]–[54]. In this regard, the psychometric identification of individuals at risk for psychosis, especially adolescents, must be nourished by incorporating the recent more modern measurement models as well as the new information technology. Computerized Adaptive Testing (CAT) is a relatively recent innovation that opens up new possibilities in personality and psychosis assessment. These incorporations will favor the assessment of the extended psychosis phenotype in a more objective and rigorous manner as well as the delimitation and understanding of the construct.

The main goal of this study was to develop and analyze the psychometric properties and efficiency of the CAT of schizotypy measured through the ESQUIZO-Q [24] in a representative sample of non-clinical adolescents. The results showed that the ESQUIZO-Q is a self-report with adequate psychometric properties both under Classical Test Theory (CTT) and Item Response Theory (IRT), and that it can be used for schizotypy assessment in adolescents. Likewise, the present study showed that CAT can be used for the assessment of adolescents with high scores on the latent trait (schizotypy) and that an improvement in efficiency takes place when administering the measurement instrument in its computerized adaptive version. These data support the validity of the ESQUIZO-Q for its use in adolescent populations and suggest the necessity of applying CAT in a real context.

From a strictly psychometric point of view, the essential objectives of this study were centered on exploring the psychometric properties of a hypothetical CAT for the assessment of schizotypy and, at the same time, comparing the performed estimations under the IRT framework with those obtained according to CTT. Although the results obtained in this study when comparing the efficiency of the computerized version with the paper-and-pencil ESQUIZO-Q are promising, there are at least two important limitations. First, we start with a measurement instrument originally designed to be administered according to CTT, with 51 items that aim to assess the complex construct of schizotypy [1]–[3]. The structure of this construct is multidimensional, just as described in previous factorial studies analyzing the internal structure of the ESQUIZO-Q [39]. According to this, it can be assumed that the original 51-item pool from which we start is not unidimensional and will negatively influence the psychometric functioning of the CAT, which is based on the unidimensionality assumption [55]. Despite efforts to control this limitation by exploring the possibility of using sub-pools of items grouping clearly unidimensional items according to substantive criteria, we abandoned this purpose given the reduced number of items in the item pool we initially had to work with. This would lead us to work with item banks not higher than 20 items.

It is very interesting to compare the results presented herein with previous CAT and personality studies. For example, Frobey and Ben-Porath [37] used the MMPI-2 in its adaptive version analyzing its functionality with the IRT method and the countdown method. The countdown method classifies individuals into one of two groups (elevated or not elevated) on the basis of whether they exceed or do not exceed a cut-off criterion on a given scale. The cut-off criterion is typically the raw score that corresponds to a clinical elevation (e.g., individuals at high risk for psychosis) on a given scale. For example, in a 10-item subscale, scoring 5 items or more in the true direction is required to reach a clinically high or significant score. It would be interesting to administer this method in the assessment of the psychotic phenotype especially when the measured construct is frequently of a multidimensional nature. In spite of these limitations, in our opinion the administration of the CAT is adequate if we consider that among the three dimensions of the ESQUIZO-Q, there is a central and dominant latent trait. In this sense, 42 items of the ESQUIZO-Q are directly related to Positive and Interpersonal Disorganization dimensions that in non-clinical adolescents are highly overlapped [39].

Second, we start with an item pool of 51 items that have the Information Function located in high levels of the latent trait (around *θ* = +2). This fact regarding the item bank hinders precise estimations both above and below this *θ* value. In this sense, only participants who score high on this latent trait will obtain reliable estimations with the CAT, whereas those participants with a lower or much higher score on the trait will exhaust the CAT item pool without reaching the appropriate termination criteria. Therefore, the *θ* estimations obtained would present serious imbalances, undermining the reliability estimations of the measurement instrument. Previous studies have found similar results when estimations of schizotypy are conducted under the IRT viewpoint [27], [56]. For example, Kin et al., [56] using the Magical Ideation Scale (MIS) in a sample of Korean adolescents, found that the MIS scores provided greater information for high scores on the schizotypy latent trait. Similar results are found when the information functions are analyzed with Wisconsin Schizotypy Scales in young adults [27]. This is an interesting fact as it allows us to assess young participants at high risk for psychosis with greater precision at the high end of the latent trait. Since the study of the psychosis phenotype is a very relevant issue on an international level, and considering that there are some issues of controversy and debate (e.g. attenuated psychosis syndrome), it would be interesting to incorporate these advances in applied research with the aim of improving strategies in early detection, identification, intervention and monitoring.

According to the results found in this study, there is no doubt that the polytomous CAT obtains better correlations with the total score of the classic test than those obtained by the dichotomous test. Although, a priori, this superiority was predictable, with the obtained results, we cannot clearly assert that the differences found between the dichotomous and polytomous models are real. This is due to the fact that dichotomous scores were obtained collapsing the lower (1, 2 and 3 = 0) and higher (4 and 5 = 1) categories of the polytomous responses once the polytomous test was administered. Thus, this artificial dichotomization of the ESQUIZO-Q scores sacrifices information in favor of the CAT administration process. At this point, it is worth mentioning that studies in the field of educational measurement have found that ability estimates resulting from polytomous scoring had slightly higher measurement precision than those resulting from dichotomous scoring [57].

The results of the psychometric properties of the CATs, after separating the calibration sample of the items (*n* = 2556) from the participant sample (*n* = 500) used for the administration of these CATs, seem to support the initial assumptions: the correlations found are similar to those found in the case of a single sample. In our opinion, the reason for this fact is due to the considerable size of the initial sample which favors the invariance of the item parameters on the one hand and, on the other hand, the robustness of the assumptions that the IRT takes as a starting point (local independence, and unidimensionality) [29], [55]. The final conclusions of this study lead us to consider these CATs as an interesting alternative in the administration of the ESQUIZO-Q given the resources saved with their application [34], as well as the validity of the estimates obtained with this procedure [58].

Adolescence is an interesting period for the early detection and identification of serious mental disorders, such as in the case of psychosis, as well as for the study of risk and protection markers. Both from a clinical and a research perspective, it is of great importance to have at our disposal measurement instruments that are brief, easy and of rapid application to use as screening methods for the detection and posterior preventive intervention of participants who are at risk for psychosis. The results obtained from studies on the psychometric high-risk paradigm have important practical implications. Measuring instruments such as ESQUIZO-Q can be used as screening tools in both educational and clinical settings. The assessment of schizotypal experiences and traits could be carried out within a multi-step process. In the first phase, we would detect those participants with a hypothetical liability to schizophrenia-spectrum disorders, based on self-report scores. Then, in a second step, there would be a more comprehensive psychological and medical assessment. In this phase, the preoccupation, conviction and stress associated to these experiences should be evaluated. Also, other risk factors of psychosis such as affective dysregulation, genetic liability (e.g. first-degree relatives of patients), cannabis use, trauma, or coping strategies would be evaluated. At this stage, information from other sources close to the individual, such as parents, close friends or teachers, can also be gathered in a multi-informant assessment. Finally, participants could be sent to a specialized mental health care center to receive prophylactic treatment. This multi-step process is just one possible way, as it may be in the case of a participant who is sent directly to a mental health care center due to the severity of his/her psychopathological signs and symptoms.

The results found in the present study should be interpreted in light of the following limitations. First, the sample used in this study corresponds to Spanish adolescents and, thus, caution should be exercised when generalizing the data to other populations of interest. Second, the extracted conclusions are founded exclusively on a self-report and there is no doubt that the use of external informants such as parents or teachers via hetero-reports would have been interesting. Third, the study developed here is a simulation for which the results found should be understood as approximations. Fourth, schizotypal experiences must always be analyzed within a biopsychosocial model. The additive or synergic interactions between schizotypal experiences and genetic, chemical, cognitive and social factors are relevant and interesting with a view to understanding and explaining the transition to the clinical disorder [11]. Fifth, no information was gathered regarding the participantś psychiatric morbidity or the use or abuse of substances, aspects that may be partially modulating the obtained results.

In conclusion, this study indicates that there is a potential for the development of adaptative tests for psychosis phenotype assessment; however, there is still much work to be done. With respect to the possible future application of CAT for the assessment of schizotypy, multiple and interesting lines of research are opened. One of these is oriented toward exploring the frequency of the presentation of items in the CAT with the aim of advancing, if possible, in the optimization of its functioning. Another is to investigate the possibility of using the computer adaptive procedure as an initial screening test in the general adolescent population.

## Supporting Information

Supporting Information S1 Items of the Oviedo Schizotypy Assessment Questionnaire (ESQUIZO-Q).
(DOCX)

## References

[1] Raine A (2006) Schizotypal personality: neurodevelopmental and psychosocial trajectories. Annual Review of Clinical Psychology 2: 291–326. 10.1146/annurev.clinpsy.2.022305.095318 17716072

[2] Lenzenweger MF (2010) Schizotypy and schizophrenia: The view from experimental psychopathology. New York: Guilford Press;

[3] Kwapil TR, Barrantes-Vidal N (In press). Schizotypal Personality Disorder: An Integrative Review. In: Widiger TA, editor. The Oxford Handbook of Personality Disorders. New York: Oxford University Press.

[4] Poulton R, Caspi A, Moffitt TE, Cannon M, Murray R, et al (2000) Children’s self-reported psychotic symptoms and adult schizophreniform disorder: a 15-year longitudinal study. Archives of General Psychiatry 57: 1053–1058. 11074871 10.1001/archpsyc.57.11.1053

[5] Welham J, Scott J, Williams G, Najman J, Bor W, et al (2009) Emotional and behavioural antecedents of young adults who screen positive for non-affective psychosis: a 21-year birth cohort study. Psychological Medicine 39: 625–634. 18606046 10.1017/S0033291708003760

[6] Gooding DC, Tallent KA, Matts CW (2005) Clinical status of at-risk individuals 5 years later: Further validation of the psychometric high-risk strategy. Journal of Abnormal Psychology 114: 170–175. 15709824 10.1037/0021-843X.114.1.170

[7] Werbeloff N, Drukker M, Dohrenwend BP, Levav I, Yoffe R, et al (2012) Self-reported attenuated psychotic symptoms as forerunners of severe mental disorders later in life. Archives of General Psychiatry 69: 467–475. 22213772 10.1001/archgenpsychiatry.2011.1580

[8] Kaymaz N, Drukker M, Lieb R, Wittchen HU, Werbeloff N, et al.. (2012) Do subthreshold psychotic experiences predict clinical outcomes in unselected non-help-seeking population-based samples? A systematic review and meta-analysis, enriched with new results. Psychological Medicine 20: 1–15. [epub ahead of print]. 10.1017/S0033291711002911 22260930

[9] Dominguez MG, Wichers M, Lieb R, Wittchen H-U, van Os J (2011) Evidence that onset of clinical psychosis is an outcome of progressively more persistent subclinical psychotic experiences: An 8-Year Cohort Study. Schizophrenia Bulletin 37: 84–93. 19460881 10.1093/schbul/sbp022 PMC3004179

[10] Kelleher I, Cannon M (2011) Psychotic-like experiences in the general population: characterizing a high-risk group for psychosis. Psychological Medicine 41: 1–6. 20624328 10.1017/S0033291710001005

[11] van Os J, Linscott RJ, Myin-Germeys I, Delespaul P, Krabbendam L (2009) A systematic review and meta-analysis of the psychosis continuum: Evidence for a psychosis proneness-persistence-impairment model of psychotic disorder. Psychological Medicine 39: 179–195. 18606047 10.1017/S0033291708003814

[12] Linscott RJ, van Os J An updated and conservative systematic review and meta-analysis of epidemiological evidence on psychotic experiences in children and adults: on the pathway from proneness to persistence to dimensional expression across mental disorders. Psychological Medicine In press. 10.1017/S0033291712001626 22850401

[13] McGorry PD, Killackey E, Yung A (2008) Early intervention in psychosis: concepts, evidence and future directions. World Psychiatry 7: 148–156. 18836582 10.1002/j.2051-5545.2008.tb00182.x PMC2559918

[14] Yung AR, Killackey E, Hetrick SE, Parker AG, Schultze-Lutter F, et al (2007) The prevention of schizophrenia. International Review of Psychiatry 19: 633–646. 18092241 10.1080/09540260701797803

[15] Fonseca-Pedrero E, Paino M, Lemos-Giráldez S, García-Cueto E, Campillo-Álvarez A, et al (2008) Schizotypy assessment: State of the art and future prospects. International Journal of Clinical and Health Psychology 8: 577–593.

[16] Lenzenweger MF (1994) Psychometric high-risk paradigm, perceptual aberrations, and schizotypy: An update. Schizophrenia Bulletin 20: 121–135. 8197410 10.1093/schbul/20.1.121

[17] Kwapil TR, Barrantes Vidal N, Silvia PJ (2008) The dimensional structure of the Wisconsin schizotypy scales: Factor identification and construct validity. Schizophrenia Bulletin 34: 444–457. 17768308 10.1093/schbul/sbm098 PMC2632435

[18] Kelleher I, Harley M, Murtagh A, Cannon M (2011) Are screening instruments valid for psychotic-Like experiences? A validation study of screening questions for psychotic-like experiences using in-depth clinical interview. Schizophrenia Bulletin 37: 362–369. 19542527 10.1093/schbul/sbp057 PMC3044617

[19] Chapman JP, Chapman LJ, Kwapil TR (1995) Scales for the measurement of schizotypy. In: Raine A, Lencz T, Mednick SA, editors. Schizotypal Personality. New York: Cambridge University Press; p. 79–106.

[20] Raine A (1991) The SPQ: A scale for the assessment of schizotypal personality based on DSM-III-R criteria. Schizophrenia Bulletin 17: 555–64. 1805349 10.1093/schbul/17.4.555

[21] Fonseca-Pedrero E, Santarén-Rosell M, Paino M, Lemos Giraldez S (2013) Cluster A maladaptive personality patterns in a non-clinical adolescent population. Psicothema 25: 171–178. 23628530 10.7334/psicothema2012.74

[22] Walker E, Bollini A (2002) Pubertal neurodevelopmental and the emergence of psychotic symptons. Schizophrenia Research 54: 17–23. 11853974 10.1016/s0920-9964(01)00347-4

[23] Rawlings D, MacFarlane C (1994) A multidimensional schizotypal traits questionnaire for young adolescents. Personality and Individual Differences 17: 489–496.

[24] Fonseca-Pedrero E, Muñiz J, Lemos-Giráldez S, Paino M, Villazón-García U (2010) ESQUIZO-Q: Cuestionario Oviedo para la Evaluación de la Esquizotipia [ESQUIZO-Q: Oviedo Questionnaire for Schizotypy Assessment ]. Madrid: TEA ediciones SA.

[25] Kwapil TR, Ros-Morente A, Silvia PJ, Barrantes-Vidal N (2012) Factor invariance of psychometric schizotypy in Spanish and American samples. Journal of Psychopathology and Behavioral Assessment 34: 145–152.

[26] Fonseca-Pedrero E, Paíno M, Lemos-Giráldez S, Sierra-Baigrie S, Muñiz J (2011) Measurement invariance of the Schizotypal Personality Questionnaire-Brief across gender and age. Psychiatry Research 190: 309–315. 21663975 10.1016/j.psychres.2011.05.021

[27] Winterstein BP, Ackerman TA, Silvia PJ, Kwapil TR (2011) Psychometric properties of the Wisconsin Schizotypy Scales: Classical test theory, item response heory, and differential item functioning. Journal of Psychopathology and Behavioral Assessment 33: 480–490.

[28] Hambleton RK, Swaminathan H, Rogers HJ (1991) Fundamentals of item response theory. Beverly Hills, CA: Sage Publications, Inc.

[29] De Ayala RJ (2009) The theory and practice of item response theory. New York: The Guilford Press.

[30] Bartram D, Hambleton R (2006) Computer-Based Testing and the Internet. Issues and advantes. England: John Wiley and Sons.

[31] Wainer H (2000) Computerized Adaptive Testing: A Primer. Mahwah NJ: Lawrence Erlbaum Associates.

[32] Van der Linden WJ, Glas CA (2010) Elements of adaptive testing. London Springer.

[33] Abad FJ, Olea J, Aguado D, Ponsoda V, Barrada JR (2010) Deterioro de parámetros de los ítems en tests adaptativos informatizados: estudio con eCAT. [Item parameter drift in computerized adaptive testing: Study with eCAT]. Psicothema 22: 340–347. 20423641

[34] Rebollo P, Castejón I, Cuervo J, Villa G, García E, et al (2010) Validation of a computer-adaptive test to evaluate generic health-related quality of life. Health and Quality of Life Outcomes 8: 147. 21129169 10.1186/1477-7525-8-147 PMC3022567

[35] Anatchkova MD, Saris-Baglama RN, Kosinski M, Bjorner JB (2009) Development and preliminary testing of a computerized adaptive assessment of chronic pain. Jorunal of Pain 10: 932–943. 10.1016/j.jpain.2009.03.007 PMC2763618 19595636

[36] Smits N, Cuijpers P, van Straten A (2011) Applying computerized adaptive testing to the CES-D scale: A simulation study. Psychiatry Research 30: 147–155. 10.1016/j.psychres.2010.12.001 21208660

[37] Forbey JD, Ben-Porath YS (2007) Computerized adaptive personality testing: A review and Illustration with MMPI-2 Computerized adaptive version. Psychological Assessment 19: 14–24. 17371120 10.1037/1040-3590.19.1.14

[38] Edelen MO, Reeve BB (2007) Applying item response theory (IRT) modeling to questionnaire development, evaluation, and refinement. Quality of Life Research 16: 5–18. 17375372 10.1007/s11136-007-9198-0

[39] Fonseca-Pedrero E, Lemos-Giráldez S, Paíno M, Sierra-Baigrie S, Santarén-Rosell M, et al (2011) Internal structure and reliability of the Oviedo Schizotypy Assessment Questionnaire (ESQUIZO-Q). International Journal of Clinical and Health Psychology 11: 385–402.

[40] American Psychiatric Association (2000) Diagnostic and Statistical Manual of Mental Disorders (4 th ed revised). Washington, DC: APA.

[41] Meehl PE (1962) Schizotaxia, schizotypy, schizophrenia. American Psychologist 17: 827–838.

[42] Fonseca-Pedrero E, Paino M, Lemos-Girádez S, Sierra-Baigrie S, Ordóñez N, et al (2011) Early psychopathological features in Spanish adolescents. Psicothema 23: 87–93. 21266147

[43] Fonseca-Pedrero E, Lemos-Giráldez S, Paino M, Villazón-García U, Muñiz J (2009) Validation of the Schizotypal Personality Questionnaire Brief form in adolescents. Schizophrenia Research 111: 53–60. 19342199 10.1016/j.schres.2009.03.006

[44] Samejima F (1969) Estimation of ability using a response pattern of graded scores. (Psychometrik Monograph 17). Richmond, VA: Psychometric Society.

[45] Birnbaum A (1968) Some latent trait models and their use in inferring an examinee's ability. Reading, M.A: Addison-Wesley.

[46] Zumbo BD, Gadermann AM, Zeisser C (2007) Ordinal versions of coefficients alpha and theta for Likert rating scales. Journal of Modern Applied Statistical Methods 6: 21–29.

[47] Markon KE, Chmielewski M, Miller CJ (2011) The reliability and validity of discrete and continuous measures of psychopathology: A quantitative review. Psychological Bulletin 137: 856–879. 21574681 10.1037/a0023678

[48] Thissen D (1991) MULTILOG, multiple categorical item analysis and test scoring using Item Response Theory. Chicago, IL: Scientific Software Inc.

[49] Walter OB (2010) Adaptative Test for Measuring Anxiety and Depression. In: New York: Springer Science+Business Media W.J. van der Linden, C.A.W Glas, editors. Elements of Adaptative Testing. LLC: 123.

[50] Statistical Package for the Social Sciences (2006) SPSS Base 15.0 User's Guide. Chicago, IL: SPSS Inc.

[51] Muraki E, Bock RD (1996) PARSCALE. IRT based test scoring and items analysis for graded open-ended exercises and performance tasks. Chicago, IL: Scientific Software International.

[52] Brennan RL (2006) Educational Measurement. Washington, DC: American Council on Education/Praeger.

[53] Hambleton RK (2004) Theory, methods, and practices in testing for the 21st century. Psicothema 16: 696–701.

[54] Olea J, Abad F, Barrada JR (2010) Tests informatizados y otros nuevos tipos de tests [Computerized tests and other new types of test]. Papeles del Psicólogo 31: 94–107.

[55] Van der Linden WJ, Hambleton RK (1997) Handbook of Modern Item Response Theory. Nueva York: Springer.

[56] Kim Y, Chang JS, Hwang S, Yi JS, Cho IH, et al. Psychometric properties of Peters et al. Delusions Inventory-21 in adolescence. Psychiatry Research In press. doi:pii: S0165-1781(12)00476-3. 10.1016/j.psychres.2012.09.002. 10.1016/j.psychres.2012.09.002 23122557

[57] Jiao H, Liu J, Haynie K, Woo A, Gorham J (2012) Comparison between dichotomous and polytomous scoring of innovative items in a large-scale computerized adaptive test. Educational and Psychological Measurement 72: 493–509.

[58] Rebollo P, García-Cueto E, Zardaín P, Cuervo J, Martínez I, et al (2010) Desarrollo del CAT-Health, primer test adaptativo informatizado para la evaluación de la calidad de vida relacionada con la salud en España [Development of the CAT-Health, the first adaptive computerized test (CAT) for the evaluation health-related quality of life in Spain]. Medicina Clínica 133: 241–251. 10.1016/j.medcli.2008.09.045 19560172

