# Supplementary material for: Development of a Computerized Adaptive Test for Schizotypy Assessment
Source: PLoS One. 2013 Sep 3;8(9):e73201. doi: 10.1371/journal.pone.0073201 (PMC3760882; doi:10.1371/journal.pone.0073201)
Supplement: Supporting Information S1 — Items of the Oviedo Schizotypy Assessment Questionnaire (ESQUIZO-Q). (DOCX) [file pone.0073201.s001.docx]

Supporting information

Items of the Oviedo Schizotypy Assessment Questionnaire (ESQUIZO-Q)

I believe that what appears on the radio or television has a special significance for me that my friends do not understand.

My lucky charms can even make me pass an examination.

I believe that there are people who can read the minds of others.

I hear voices that others cannot hear.

I believe that there are persons who can control the thoughts of others.

I believe that someone is plotting against me.
